# Synchronised Video, Motion Capture and Force Plate Dataset for Validating Markerless Human Movement Analysis

**DOI:** 10.1038/s41597-024-04077-3

**Published:** 2024-11-28

**Authors:** Murray Evans, Laurie Needham, Logan Wade, Martin Parsons, Steffi Colyer, Polly McGuigan, James Bilzon, Darren Cosker

**Affiliations:** 1 Centre for the Analysis of Motion, Entertainment Research and Applications, University of Bath, Bath, UK; 2 Department of Computer Science, University of Bath, Bath, UK; 3 Department for Health, University of Bath, Bath, UK; 4 Seiko Timing Systems, Berkshire, UK; 5 Microsoft, London, UK

**Keywords:** Scientific data, Musculoskeletal system, Data acquisition, Image processing, Biological physics

## Abstract

The BioCV dataset is a unique combination of synchronised multi-camera video, marker based optical motion capture, and force plate data, observing 15 healthy participants (7 males, 8 females) performing controlled and repeated motions (walking, running, jumping and hopping), as well as photogrammetry scan data for each participant. The dataset was created for the purposes of developing and validating the performance of computer vision based markerless motion capture systems with respect to marker based systems.

## Background & Summary

Marker-based optical motion capture for measuring human motion is well established but there remain issues that limit the scope of the technique^1,2^. Correctly placing markers on the participant can be time consuming, markers may fall off or impede the natural performance of the participant, and the markers move with the skin rather than the bones beneath (the “skin motion artifact”^3^). Furthermore, the camera systems used to track the markers can be expensive and often limited to laboratory conditions.

Advances in computer vision and artificial intelligence systems have lead to an explosion in techniques^4–6^ for markerless human motion capture. If these techniques can be used to make accurate biomechanical measurements of humans it could have a major impact on biomechanical research studies and applications. Going markerless could enable the collection of measurements with reduced equipment, in time limited clinical settings, elite sports training and competition, or “in the wild”.

The BioCV dataset was created to facilitate the development and validation of markerless motion capture techniques in the domain of human biomechanics. The dataset consists of:

1. calibrated 9-camera synchronised HD colour video at 200 Hz
2. 3D marker trajectories from optical motion capture, time synchronised to the videos
3. synchronised analogue force plate data.
4. 15 human participants (7 female, 8 male) each performing repeated trials of the same specific movements (walking, running, jumping and hopping)
5. photogrammetry scans of each participant
6. primary trials with synchronised video and markers
7. secondary markerless trials with video data only and no markers.

The dataset has some similarity with previous datasets such as HumanEva^7^ and Human3.6M^8^, however the specific controlled motions, biomechanically-robust marker positions and inclusion of force plate and photogrammetry data make it unique. Currently there are no plans to expand upon this dataset.

Human biomechanics is the study of how people move and how those movements affect health and performance. A significant part of that is understanding how forces propagate through the body to produce movement. Force plate data is commonly obtained in laboratory environments, however its use in real world applications is generally more limited. As such, predicting forces and joint moments from motion capture data is an active area of research^9–11^, and the provision of force plate data in this dataset should aid in developing and validating both marker-based and markerless approaches.

It is common to fit human body models to the observations of motion capture systems when making pose measurements. The photogrammetry data is intended to help facilitate the creation of person specific body models, and evaluate whether such models improve the performance of body tracking algorithms.

A number of publications used a pre-publication version of the dataset. The final, published dataset differs only by 1) cropping the video and motion capture data to remove periods of inactivity at the start/end of trials, and to align the marker trajectory files such that there is no need for external time offset information, and 2) provide an updated calibration for the machine vision system. Conclusions of published papers should remain the same but their results may not be exactly reproducible even if using the original calibrations. The relevant publications were all conducted within the *Centre for the Analysis of Motion, Entertainment Research and Applications* (CAMERA) at the University of Bath:

1. “Examination of 2D frontal and sagittal markerless motion capture: Implications for markerless applications”, Wade *et al*.^12^
2. “Backward Double Integration is a Valid Method to Calculate Maximal and Sub-Maximal Jump Height”, Wade *et al*.^13^
3. “The Development and Evaluation of a Fully Automated Markerless Motion Capture Workflow”, Needham *et al*.^14^
4. “The Accuracy of Several Pose Estimation Methods For 3D Joint Centre Localisation”, Needham *et al*.^15^
5. “Development and Evaluation of a Deep Learning Based Markerless Motion Capture System”, Needham *et al*.^16^
6. “The Performance of Open-Source Pose Estimation Algorithms During Walking, Running and Jumping”, Needham *et al*.^17^

The above papers describe the development and validation of a 3D markerless motion capture pipeline based on modern “sparse keypoint” pose detectors, validation of the 2D keypoint detections, and validation of a marker based approach to jump height estimation.

To further support the dataset, software is provided for camera calibration, a 3D markerless motion capture pipeline, and related visualisation tools. The tools are available through a Github repository (https://github.com/camera-mc-dev)^18^.

## Methods

Fifteen human participants consented to take part in the dataset, and each completed a set of motion trials and a separate photogrammetry scan. The motion trials were measured by 3 sensor systems:

1. 200 fps HD colour video from a 9 camera machine vision system
2. markers tracked with a 15 camera optical motion capture system synchronised to the camera system.
3. analogue signals from force plates embedded in the floor.

To maximise the value of the data, great care has been taken to ensure that the different data sources have high quality calibrations and are time synchronised and spatially aligned.

Photogrammetry (in this context) refers to the process of reconstructing detailed 3D representations of surfaces from images. The intention is that the resulting 3D reconstructions can be used to create personalised body models or infer relevant measurements about each participant which could be used by candidate motion capture systems to improve their tracking performance.

### Participants and Movements

The BioCV dataset consists of 15 participants, with each participant undertaking repeated trials of the same base movements. Each participant performed 10 running trials at a self-selected speed, 10 walking trials at self-selected speed, 10 counter movement jumps (five maximal and five sub-maximal effort) with hands held on the waist and 10 two-footed hops. The movements are based on those commonly used for biomechanical assessments of the lower limbs. The available data thus facilitates determining the general accuracy of pose estimation systems, as well as whether there is sufficient accuracy to identify the differences between individual people, and variation of one person between different recordings. Previous data sets Human Eva and Human3.6m have been repeatedly used to perform validation on pose estimation methods while only encapsulating data collection of four and eleven people respectively. As such, this sample size of participants exceeds both of these previous datasets. Crucially, the current dataset includes repeated trials of the same movement, enabling the assessment of pose estimation methods to accurately identify posture within the same person. We do note the limited range of activities included in this dataset, which may limit the use of this dataset for some applications. These movements were chosen as the goal of this dataset was to facilitate development of pose estimation methods targeted at improving identification of lower limb kinematics, which are crucial for biomechanical applications that require accurate detection of pose at an individual person level.

The participants were healthy volunteers and all provided written informed consent, both to take part in the experiments and to have video/image data of themselves included in a public dataset. Data were collected for 7 males [1.82  ± 0.11 m, 85.7  ± 11.1 kg] and 8 females [1.65  ± 0.08 m, 63.2  ± 6.0 kg]).

**Fig. 1:**
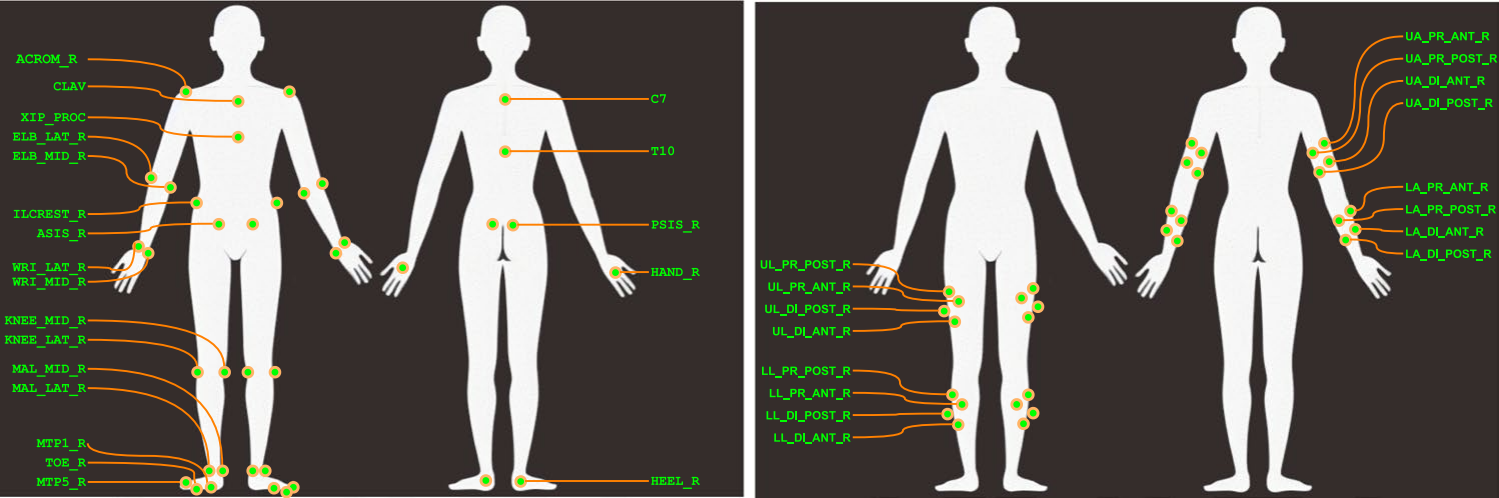
Markers (left) and marker clusters (right). For clarity, only the right side markers names have been shown (left side marker names change the ‘_R’ to ‘_L’).

In the primary trials, each participant completed all movements while wearing a full body marker set comprising of 38 individual markers and eight 4-marker clusters allowing for a full body six degrees of freedom (6DoF) model (bilateral feet, shanks and thighs, pelvis and thorax, upper and lower arms, and hands) as shown in Fig. 1. Hand markers were placed of the lateral aspect of the third metacarpophalangeal joint (base of middle finger), wrist markers were placed on medial and lateral aspects of the ulna and radius stylus processes, while elbow markers were placed on the medial and lateral epicondyle of the humerous, for both the left and right arms. Markers were placed on the the acromion process of the left and right shoulders, suprasternal notch of the manubrium, the xiphoid process, C7 and T10 vertebrae spinal process, left and right posterior superior iliac spine, left and right anterior superior iliac spine and along the left and right iliac spine directly superior to the greater trochanter. Markers were placed on both legs at the medial and lateral joint centre of rotation of the knee, medial and lateral malleolus of the ankle. Clusters of four markers on rigid plates placed on the lateral side of the humerous, forearm, shank and thigh, midway between joints using velcro straps. Markers for both feet were placed on the shoes over the posterior aspect of the calcaneus, metatarsal phalangeal joints 1 and 5, and the distal phalanx of the first toe.

A secondary set of trials was also conducted, completing one trial of all movements with no markers.

All trials for each participant were completed in a single session. Trials for a specific movement were done one-after-the-other, but the order of movements was randomised between participants. The full set of movements is shown in Table 1.

### Hardware

Tables 2, 3 and 4 identify the full list of hardware used for the machine vision, motion capture and photogrammetry systems.

**Table 1:** Complete set of movements.

tag	number	description
CMJM	5	counter movement, maximal effort
CMJS	5	counter movement, sub-maximal effort
WALK	10	straight line walk at self selected pace
RUN	10	straight line run at self selected pace
HOP	1	in place two-footed hopping on the force plates (10 hops)
ML	5	one trial of each movement *without* markers

**Table 2:** Hardware for the machine vision system.

Item	Make	Model	Quantity	Description
Camera	JAI	SP-5000C-CXP2-C	9	Machine vision cameras with CoaXPress interface
Lens	KOWA	LM6HC	7	6 mm lens used by 7 cameras
Lens	KOWA	LM8HC	2	8 mm lens used by 2 cameras
Framegrabber	SISO	MICROENABLE 5 AQ8-CXP6D	3	camera <-> computer CoaXPress interface
I/O interface	SISO	OptoTrigger 5	1	transmit timing and control signals from recording server
Recording server	Various	N/A	1	Linux based recording system configured by Stemmer. Recording software is custom by CAMERA using SISO API
Lighting				Mix of LED floodlights and bounced fill lights

**Table 3:** Hardware for the marker-based motion capture system.

Item	Make	Model	Quantity	Description
NIR camera	Qualisys	Oqus	15	Qualisys Oqus cameras for marker observation
Forceplate	Kistler	9287CA	4	Floor embedded force plates
L-frame	Qualisys		1	Qualisys L-frame for calibrating ground plane
Calibration wand	Qualisys		1	Qualisy calibration wand (Calibration 600 Kit - 601.4mm wand)
Computer			1	Windows based server with appropriate Qualisys I/O interfaces and Qualisys Track Manager v2019.3
Markers			38	IR reflective motion capture markers
Clusters			8	group of 4 IR reflective motion capture markers for segment tracking

**Table 4:** Hardware for photogrammetry system.

Item	Make	Model	Quantity	Description
Camera	Canon	EOS 1300D	50	DSLR cameras
Camera	Canon	EOS 2000D	14	DSLR cameras
Lens	Canon	EF-S18-55mm f/3.5-5.6 III	64	55 mm lenses for cameras
Computer			1	Windows based computer with Agisoft Photoscan photogrammetry software

**Fig. 2:**
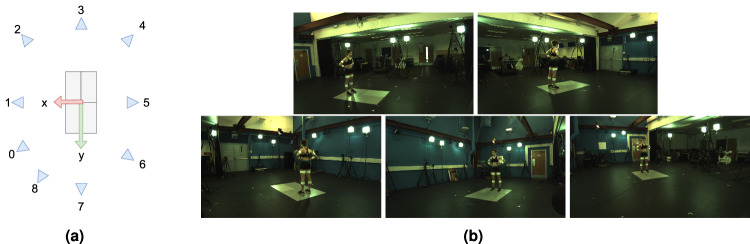
Camera layout (**a**) and example images (**b**) from even numbered cameras.

The machine vision system’s JAI cameras are capable of 5 MP resolution but were configured to output a centre crop at HD resolution (1920 x 1080, 2 MP) enabling 200 Hz capture for all 9 cameras. The cameras were arranged in a circle at about waist height and made use of wide angle lenses to maximise the capture volume for running and walking trials. Participants were approximately 500 pixels tall when standing at the centre of the volume. The cameras were positioned such that the force plates were at the centre of the capture volume. Each participant was recorded in their own session and cameras may have been moved between sessions, but care was taken to keep the arrangement equivalent between participants. A diagram of the arrangement and sample viewpoints are provided in Fig. 2.

At 200 fps, lighting is a significant concern, and as such, the laboratory was rigged with a number of LED floodlights, each of which was given a custom diffuser and allowed to directly illuminate the participants. Two bright, high wattage incandescent lights were further “bounced” via the ceiling/walls to increase fill light (the integrated room lights were dim or induced significant mains frequency flicker so were disabled).

The “framegrabbers” serve as the I/O interface between the recording computer and the cameras. The manufacturer (Silicon Software - SISO) provide an API through which custom CAMERA “grabber” software can control the cameras and capture images. Although somewhat specific to the SISO hardware, the grabber software does form part of the published software suite, see the Code Section.

The “Optotrigger” is an I/O interface in the capture computer allowing signals to be communicated between the frame grabbers and external hardware. In particular, it is used to transmit synchronisation and stop signals to the motion capture system.

### Synchronisation

The primary framegrabber of the machine vision system was configured to generate a TTL synchronisation pulse on which all machine vision system cameras would trigger a frame acquisition. As such, all 9 cameras are hardware frame synchronised within the response time of the cameras.

The TTL timing signal was routed from the primary frame grabber, through the Optotrigger board, and outward to the marker based Qualisys motion capture system. Qualisys’s capture software (Qualisys Track Manager) was set to accept this external timing signal as the trigger for its own cameras.

Custom video capture software on the machine vision system continually recorded images to system RAM, then, when a motion “trial” was completed, the system operator would press a key to pause recording and “dump” the in-memory images to disk. This stop signal propagated via the Optotrigger and into the Qualisys server to stop recording on the marker based system. As such both recordings *should* have exactly the same end point. In practice however the marker based system would have a short but inconsistent delay before it would stop leading to a slight frame offset in the data. To resolve this, an Arduino was constructed with a pair of LEDs - one visible light, one near-infrared, in series, that would flash at the same moment. The Arduino was programmed to pulse the LEDs in an identifiable pattern. The LED pulse could be captured visibly in the marker based and machine vision systems allowing for the unwanted offset to be corrected.

In a subset of the data, the LED was not visible, or not placed in the scene. Keeping in mind that both the machine vision system and marker based system had a common “stop” trigger, the average (median) number of extra end frames in the marker based system was determined. The alignment of motion capture to video was compensated using that median when the LED was not available, and visually verified.

Force plate data were recorded directly in Qualisys Track Manager, and thus synchronised to marker data automatically by that system.

### Calibration

The Qualisys motion capture system was calibrated as per manufacturer’s instructions using a standard wand and an L-Frame to set a ground plane and origin.

**Fig. 3:**
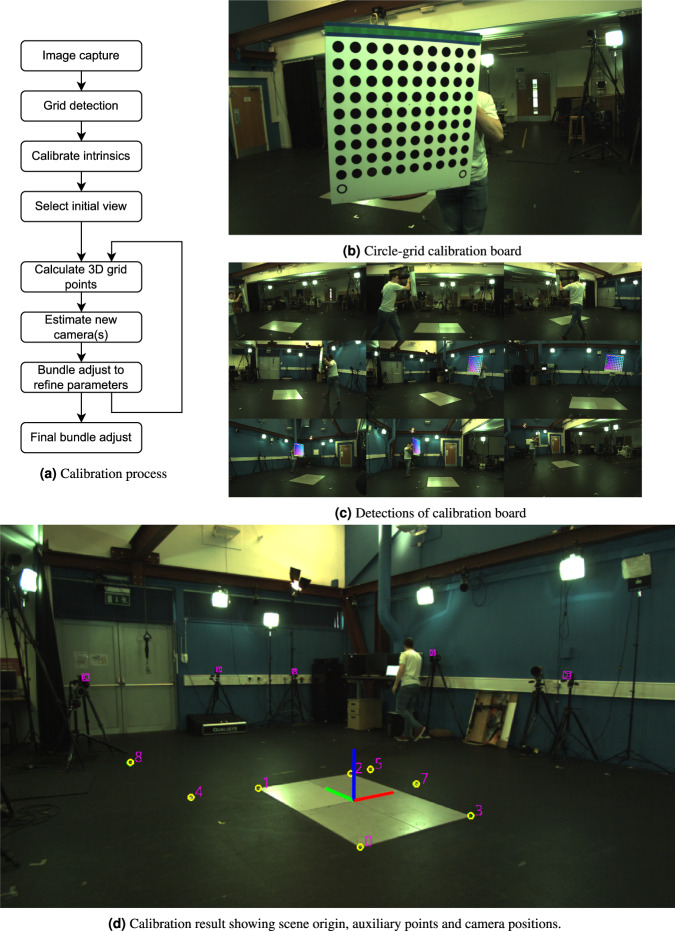
Calibration of the machine vision system.

The machine vision system was calibrated by capturing observations of a circle-grid calibration board shown in Fig. 3. The board was moved through the scene to maximise visibility to individual cameras, but critically, between pairs and triples of cameras, examples of which are seen in Fig. 3. When done correctly this creates a graph where every camera is connected (possibly indirectly) to all other cameras through shared board observations. Intrinsic camera parameters were initialised using OpenCV^19^ (which follows the approach of^20^). Extrinsic parameters are then initialised in an iterative process. One camera is selected and all grids visible from that camera have their positions estimated in 3D. The position of a second camera can be estimated based on those grid positions. The second camera allows more grid positions to be estimated, allowing more camera extrinsics to be estimated, etc... A Ceres Solver^21^ based bundle adjustment^22^ is used to optimise parameters at each stage, see Fig. 3 for a diagram of the calibration process.

Due to the calibration board being one-sided, cameras on opposite sides of the scene do not share observations of the board, which can sometimes hinder the calibration. Where necessary, extra points in the scene (such as the corners of force plates or marks on the floor) were manually annotated and included in the calibration process to help anchor the solve.

After the final bundle adjustment, a transformation is computed to place the scene origin at the ideal location to align the calibration with the motion capture system. This transformation is initially computed using manual annotations of the L-frame used for setting the motion capture system’s origin.

To further ensure alignment between the two systems, a single marker was moved through the capture volume. The marker was manually tracked in the machine vision system, and automatically tracked by the marker based system. The resulting observations were used to solve for a transformation that minimised the error between observations.

Figure 3 shows an example calibration, with scene origin, projections of camera locations, and auxiliary annotations.

During final validations of the dataset (see Validation section), a further refinement of the alignment was deemed possible. This was performed using annotations of multiple markers on each RUN_01 trial, and benefited from a better solver and better temporal alignments. Due to the late stage at which this alignment was conducted, the updated calibrations are provided in a separate download: calibrationUpdate.tar. Use of the updated calibration is recommended - the original calibration remains in the dataset for consistency with previous publications.

The time synchronisation and spatial alignment of the two capture systems was validated as shown in the Validation Section.

All software for calibration is provided in the tools accompanying the dataset (see the Code Section).

### Motion Capture Processing

The marker trajectories were labelled and gap-filled by the capture software (Qualisys Track Manager), then exported to Visual 3D (v6, C-Motion Inc), where raw trajectories were low-pass filtered (Butterworth 4th order, cut-off 12 Hz) and a 6DoF model was computed. Joint centres were computed as the point 50% between the medial and lateral markers for the ankle, knee, elbow and wrist joints, with the hip joint centres computed using the regression equations reported by Bell *et al*.^23^, the shoulder joint centres computed as being inferiorly offset by 2.5 cm from the acromioclavicular joint markers, and the MTP joint as the midpoint between the first and fifth MTP joint markers. The non-standard definition of shoulder joint centre was performed to simplify this joint, given this dataset was designed to assess lower limb kinematics and as such, have little reference at the shoulder joint. We note that if shoulder kinematics are of interest to future users, then analysis should be performed using raw .c3d files using the authors own biomechanical model.

Visual 3D’s automatic_gait_events function^24^ was applied for walking and running trials to compute touch-down (TD) and toe-off (TO) events. In the event files, RON/LON/ROFF/LOFF refer to TD and TO events obtained from the force plate data, while RHS/LHS/RTO/LTO refer to TD and TO events obtained from motion capture data. Jumping trial event data was also obtained from first movement (First_Movement) to stabilisation after landing (Stable). Where first movement was defined as the point that the vertical force dropped below body weight for 20 consecutive frames during the countermovement and stabilisation was defined as the point that vertical force remained within 3 standard deviations of bodyweight after landing. Two versions of the event files for each trial are present that provide information relative to time (seconds) and frame number. In all processing, force plate data were zeroed at the start of each trial, prior to the participant standing on the plate, with no other pre-processing of force plate data performed to obtain gait event data.

The dataset provides two .c3d files as described in the Data section. The first (raw.c3d) contains just the raw marker trajectories. The second (markers.c3d) provides the processed marker trajectories and estimated joint centre locations. Gait events are provided in two CSV files. A full list of markers is provided in a spreadsheet file (MarkerNames.xlsx), which also shows whether the marker originates in the raw data or is a processing output.

### Photogrammetry

Photogrammetry “scans” were captured using an array of 64 DSLR cameras which could be collectively controlled and triggered using *CaptureGrid* (https://kuvacode.com/). The images were used as inputs for the software *Agisoft: Photoscan* which provided “point cloud” reconstructions.

**Fig. 4:**
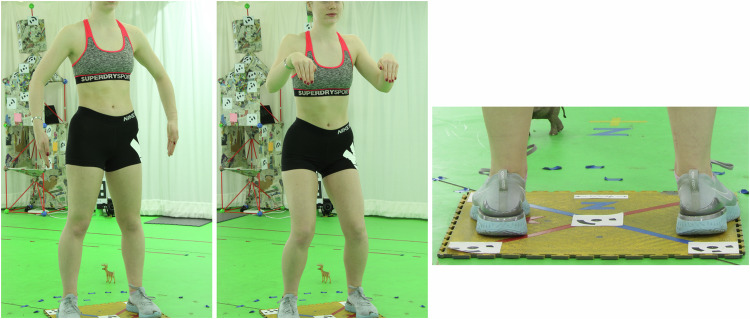
Example A-pose and C-pose used during photogrammetry scans, and view of the floor plate and markers used for scaling.

Each scan was conducted with the participant standing on the same plate, set flat on the floor at the centre of the volume. This plate had five visual markers affixed to its top side - one central, and 4 at the corners. The 5 visual markers are thus centred on the coordinates (0, 0, 0), (200, 200, 0), (−200, 200, 0), (200, −200, 0), (−200,−200, 0) (measurements in mm). The floor plate and its markers can be seen in the final image of Fig. 4.

Participants were asked to provide 2 scans. The first is a standard A-pose (upright, feet apart, legs straight, arms straight, arms and hands separated from body) as well as what was termed a C-pose, which aimed to make joint positions more identifiable by inducing small bends in ankles, knees, hips, hands, wrists, elbows, etc. For the A-pose, hair was generally visible, while for the C-pose scan, hair was covered by a patterned hat. Examples can be seen in Fig. 4.

Photogrammetry data for several participants also includes a scan of a calibration target. This target was sometimes more reliable for initialising camera positions and is included incase it is useful.

### Ethics

The study was conducted according to the guidelines of the Declaration of Helsinki, and approved by the Institutional Review Board (or Ethics Committee) of the University of Bath (EP1819052 25/07/19).

## Data Records

The BioCV dataset^25^ is made available through the University of Bath Research Data Archive at https://researchdata.bath.ac.uk/id/eprint/1258.

The dataset is divided into 17 archives (in the common .tar format), one for each of the 15 participants, one containing updated calibration files, and one containing notes detailing missing markers.

Each participant’s archive has a common structure, consisting of a root directory containing calibration information for the machine vision cameras, and a directory for each motion trial. Within the motion trial directories are the videos from 9 cameras and marker trajectory files. Table 5 shows the overall structure.

**Table 5:** general structure of the dataset.

participant	directories	motion trials	photogrammetry
P03	calib_00/	9 .mp4 video files	a.obj
P04	photogrammetry/	3 .c3d marker files	c.obj
P06	2x 9 .calib files	2 event files	a/ (images of a-pose)
P08	37 trial directories		c/ (images of c-pose)
P09			t/ (images of calib target)
P10			
P13			
P16			
P17			
P18			
P19			
P24			
P26			
P27			
P28			

At the top level are the participant directories, each containing the directories shown. The final columns show the content of each trial directory, and each photogrammetry directory

An update of the calibrations for the machine vision system was created during final validation of the dataset as described in the Validation section. The calibrationUpdate.tar archive contains these calibrations.

When processing the marker data, notes were made of instances where there were missing markers. Those notes are included in the markerNotes.tar archive.

Finally, the file participantData.csv contains height and mass information for each participant, and the MarkerNames.xlsx file describes each marker.

### Motion trials

Each motion trial exists in a single directory beneath the participant directory. Primary motion trials contain 9 video files, 3 motion capture files, and 2 events files. There are also a set of markerless motion trials, conducted without markers. These secondary markerless trials contain only the 9 video files.

Video files are numbered from 0 to 8 and the number will correspond to the calibration file in the participant’s directory. Each video file was encoded using FFMPeg and is an MPEG4 container with an h265 video stream that uses yuv444p colour format. The videos can be played by most video players, though the yuv444p colour format may cause issues with a few common tools. FFMpeg and VLC are known to work. Practitioners in the field of computer vision should have no difficulty using the videos through OpenCV with the FFMPeg backend, as is common practice.

Marker-based Motion capture data is provided in the .c3d file format which has become something of a standard for marker trajectory files. Libraries such as EZC3D^26^ have been used reliably with the files of this dataset. Three files are provided for all primary motion trials:

1. raw.c3d: “raw” unprocessed marker trajectories as output from Qualisys Track Manager.
2. markers.c3d: marker trajectories with joint centres, processed as described in the Methods section.
3. led.c3d: “trajectory” of the flashing LED used for auxiliary time alignment. If this file is missing, then the trial has an approximate temporal alignment (see Methods section).

Motion capture data and video data have been cropped and aligned such that frame *n* of the video corresponds to frame *n* of the motion data (assuming video and motion processing tools used by the user have a consistent 0-based or 1-based indexing). Marker labelling for walking and running was performed from heel-strike of the step preceding the first stance on the force plate, until toe-off of the step following the last stance phase on the force plate. For jumping and hopping, the entire trial was labelled. Missing trajectories up to 10 frames long were gap filled using spline filling in Qualisys Track Manager, with larger gaps left unfilled. Detailed notes for every trial, outlining marker gaps and sizes that occurred within this period (especially gaps when in contact with the force plate) are available in the markerNotes.tar file.

The motion events identified by Visual3D are provided in two files: markers.events.frames and markers.events.time which both show the same data, but enumerate time using either a frame number or time in seconds. The files are tables, with each column identifying a specific type of event, and the rows indicating the times at which that event type was observed. The structure should be obvious when opened in a text editor, with the first few lines showing metadata for each column, and remaining lines containing data.

### Calibration

One calibration trial is provided for each participant, and is named calib_00. The trial directory contains the 9 video files used for calibration of the machine vision system as well as the 9 .grid files which detail the detected grids.

There are *two* sets of calibrations provided for each participant. The first calibration is consistent with previous publications, while the second calibration improves upon the alignment between machine vision system and marker based motion capture system. For details of this improvement see the Validation section.

The first set of calibration files are included in the .tar files of each participant. The updated calibration files are contained in a separate .tar file - calibrationUpdate.tar. It is recommended to use the updated calibrations.

Camera calibration files consist of:

1. captured image size (width and height)
2. 3 × 3 matrix of intrinsic parameters *K*
3. 4 × 4 extrinsic matrix *L* - this is a homogeneous transformation matrix such that *p*_*c*_ = *Lp*_*w*_ takes a point in world coordinate system *p*_*w*_ and transforms it into camera coordinate system *p*_*c*_.
4. 5 distortion parameters *k* = *k*_0_, … , *k*_4_ as 3 radial distortion parameters and 2 tangential distortion parameters [*r*^2^, *r*^4^, *t*_0_, *t*_1_, *r*^6^], consistent with OpenCV^19^ and with the Bouguet calibration toolbox^27^.

These are written to a simple text file of the format:

<img width> <img height>

K00 K01 K02

K10 K11 K12

K20 K21 K22

L00 L01 L02 L03

L10 L11 L12 L13

L20 L21 L22 L23

L30 L31 L32 L33

k0 k1 k2 k3 k4

Files containing grid detections are also simple text files, which indicate a frame number, and how many grid points were detected in that frame (one grid is assumed per frame). If a grid is detected, then each detected point is enumerated with its grid row, grid column and image coordinates (*x*, *y*).

<frame no.> <num points>

<row> <col> <x> <y>

### Photogrammetry

Within the photogrammetry/ directory of each participant, are 2 point clouds (a.obj,b.obj) and 2 image directories (a/,b/). The image directories contain the 64 camera images for the ‘A’ and ‘C’ poses and the point cloud files the respective reconstructions. Image data is provided on the assumption that modern and future technology may provide better point clouds than the ones provided.

**Fig. 5:**
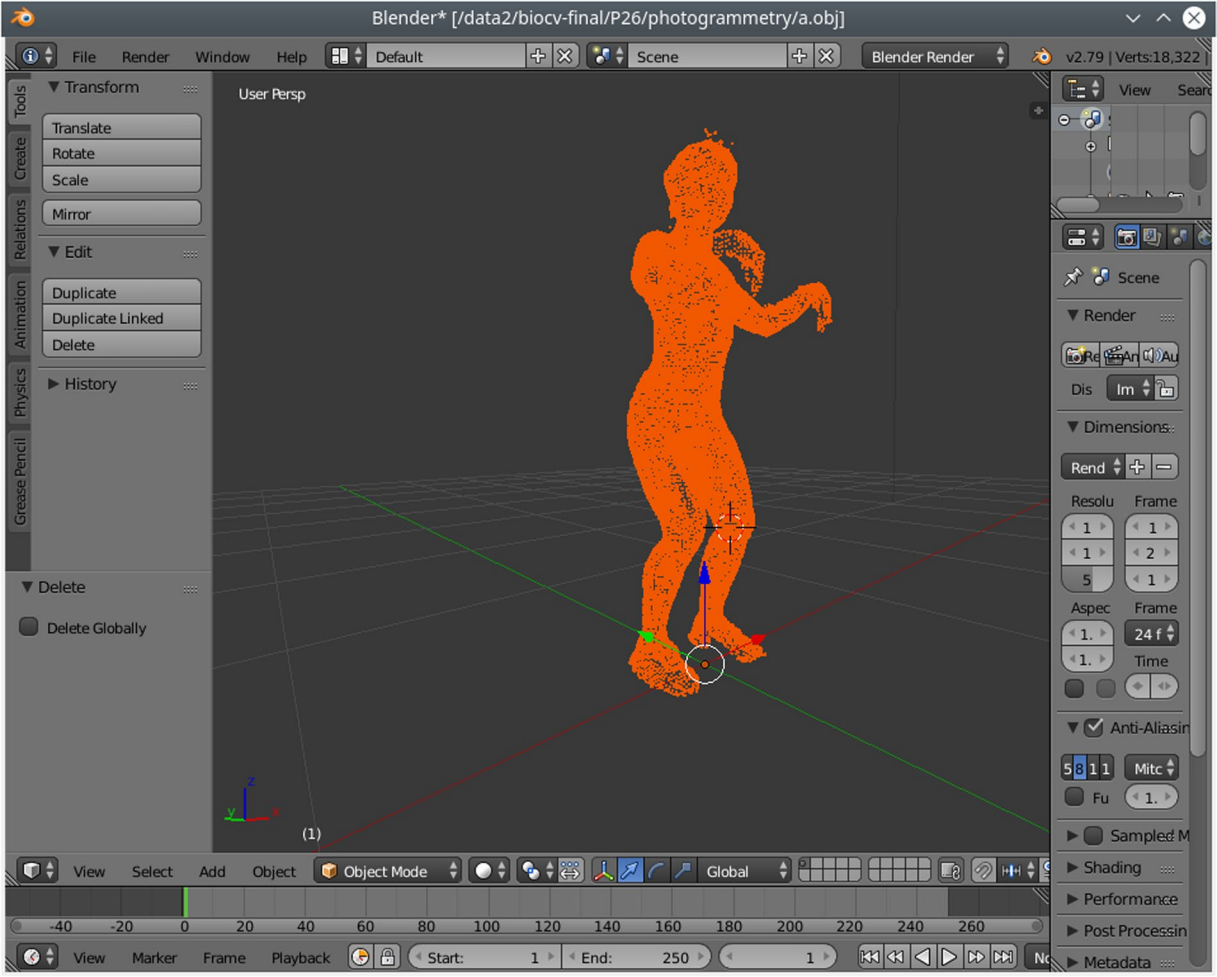
Photogrammetry point cloud reconstruction shown in Blender.

The point clouds are provided in the industry standard Wavefront OBJ format which can be imported into almost all 3D visualisation and editing packages. There are many software libraries which can load the data for the users’ preferred programming language. Figure 5 shows a point cloud in the popular 3D modelling tool Blender.

### Missing data and known problems

The trial P04_WALK_05 has missing video data (the videos were corrupted after capture), however motion data are still available.

The trial P28_RUN_10 is missing.

A number of videos for participant P08 have video capture errors - artifacts caused by missing frames where the video may seem to jump momentarily to an earlier time. Motion data are unaffected.

## Technical Validation

The primary aspect of the dataset that required validation was the temporal and spatial alignment of the machine vision system and the motion capture system. The spatial resolution of the machine vision system can also be estimated.

Temporal misalignment should have been minimised by the hardware synchronisation methods described in the Methods section, while spatial alignment depends on the quality of manual marker annotations.

### Spatial Resolution

Spatial Resolution is here defined as “how far must a point move to register a 1 pixel change in position in an image?”

To answer this question, points were initialised at the corners and centres of a cuboid spanning from (-750,-1500,0) to (750,1500,2000), which is the primary “action area” of the scene above the force plates. Each point is then moved on a line parallel to the image plane of each camera until its projection has moved 1 pixel.

On average, the points must move 4 mm to cause a 1 pixel change in projection. Note, of course, that this will change significantly for points much nearer or further from the camera, but is relevant to give context to the errors reported in the following validations.

### Initial Validation using original calibrations

Markers (ACROM_L, ACROM_R, KNEE_LAT_L, KNEE_LAT_R) were annotated in multiple views every 100 frames in each of the RUN_01 trials. Furthermore, annotations were made of the TOE_L and TOE_R markers when the relevant foot was in contact with the ground, at mid stance when the marker would be most static. The annotations were used to create a set of 3D points *A* which could be compared against the corresponding coordinates *M* reported by the marker based motion capture system.

By using the toe markers at mid-stance, spatial alignment can be validated in isolation from temporal alignment, because the markers are effectively static. The mean distance between *A* and *M* for the toe markers was computed as 6.7 mm.

**Fig. 6:**
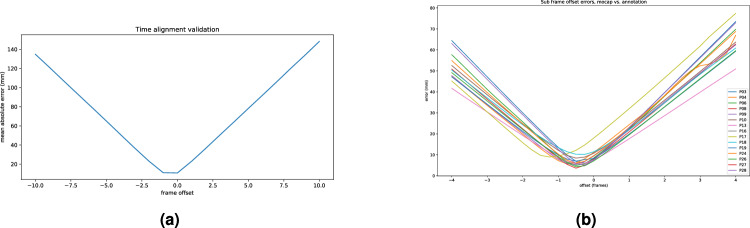
Time alignment errors at whole frames (**a**) and sub frame (**b**) leading to the conclusion that the marker based system had a slight delay vs. the machine vision system.

Temporal alignment can be validated by computing the mean distance between corresponding points at multiple frame offsets. If temporal alignment is correct, the minimum of this error should be found at an offset of 0 frames. The ACROM and KNEE markers were used for this as they are in constant motion, so a time offset will induce a distance error. Figure 6 shows the errors for each participant as the time offset is varied from -10 to 10 frames. In almost all cases, there is an ambiguous minimum for an offset of -1 or 0 frames, which suggested a sub-frame offset. To further test this, the marker trajectories were interpolated using a spline fit (SciPy, make_interp_spline) and compared to annotations at sub-frame offsets. The result, (shown in Fig. 6(b)) confirms that the ideal temporal alignment appears to be between video frames. This implies that despite the use of hardware triggers, the Qualisys motion capture system did not capture frames at the exact time of the trigger. In most cases this is unlikely to result in a major error but worthy of acknowledgement.

Overall, across all annotated markers, the calibration was found to have a mean absolute error of 6.41 mm at best sub-frame offsets. Above the force plates where most of the observed activity takes place, the error is smaller: 3.8 mm, while the average outside this area is nearer 8 mm. At the extremes of the volume the misalignment can be several pixels and thus visible.

Annotating the markers accurately in the centre of the scene is generally easy, but towards the edges of the scene can become significantly harder due to extremes of scale (markers can be large enough to make the centre ambiguous, or far enough away to be barely visible). While that can account for some of the increased error, the visible misalignment at the edges of the scene suggested updating the calibrations to possibly improve the alignment.

### Second Validation

The annotations in the first validation were used to compute a new transformation consisting of rotation, translation and scaling, that would minimise the distance between *A* and *M*. After applying this transformation to the calibrations and recomputing the 3D point for the annotations, the mean distance between annotation and marker sets was reduced to 4.3 mm overall (2.95 mm above the force plates, 5.16 mm elsewhere).

To further validate, annotations were made on the WALK_01 trial of each participant. The annotations consisted of the same subset of markers but were only made on three frames: A mid-stance frame about 1 m before the force plates, a mid-stance frame near the centre of the force plates, and a mid-stance frame about 1 m after the force plates.

The distance error for the original and updated calibrations for these annotations were 6.34 mm (4.6 mm above the forceplates, 7.17 mm elsewhere) and 3.62 mm (2.31 mm above the forceplates, 4.17 mm elsewhere) respectively.

The updated calibration is a clear improvement over the original calibrations, but the change is small enough in the centre of the volume that the conclusions of previous works should not be seriously affected.

## Usage Notes

The dataset consists primarily of video data, image data and motion capture data. The Data section provides suggested tools for basic viewing of the videos, and suitable programming APIs for working with the motion data.

A number of software tools have been created alongside this dataset, details of which can be found in the Code section.

### Accessing the data

Users of the dataset can do so through the University of Bath Data Archive: https://researchdata.bath.ac.uk/id/eprint/1258, where they will be presented with information about the dataset and a button to request access. Access to the data is unlikely to be denied - the request process exists to satisfy ethical requirements; ensuring potential users have been made aware of their responsibilities regarding the use of video containing people (namely, to treat it respectfully and to not misuse the data in a way that could be considered demeaning or which could embarass the participants), and that the data is to be used only for its intended purpose (the validation and development of markerless and marker based motion capture technologies).

### Preparing the data

The recommended approach is for users of the dataset to first create a directory biocv on their computer. They can then download the desired participant archives *and* the calibrationUpdate.tar archive to this biocv directory. The archives should then be extracted in place, this will put each participant in a directory, and write the updated calibrations to each participant directory.

### Markerless Human Motion Capture

The data is primarily suited to validating the performance of systems for markerless human motion capture. This section will briefly discuss the methods by which markerless motion capture can be undertaken - the technologies and tools are under rapid research and development and while there are a number of more established methods, there are few “off the shelf” tools that can deliver the full process of video-to-body-measurements in an end-to-end way.

A number of paradigms for markerless human pose estimation from video are possible:

1. Sparse Keypoints
2. Coarse segmentation
3. Dense Segmentation
4. Direct model fits
5. Volumetric approaches

Sparse Keypoint approaches to markerless motion capture are the most common and developed: A set of keypoints commonly corresponding to joint centre locations are detected in the image, fused across views to create 3D keypoints, and then body models suitable for biomechanical measurement are fit to the data. The sparse keypoints are often detected using popular algorithms such as OpenPose^28^ or AlphaPose^29^, but a wealth of detectors now exist. A useful starting point for researchers is the MMpose library^30^ which provides a range of modern detectors. These detectors should be compatible with the tools described in the Code section and in our previous publication^16^, but other “fusion” pipelines for consideration are OpenCap^31^ and Pose2Sim^32^. It may also be possible to use this dataset with commercial software such as Theia3D (Theia Markerless, Inc), which appears to take a similar sparse keypoint approach.

End to end markerless motion capture systems could also be based on coarse or dense body part segmentation. These tools typically are still based on 2D processing of the image data followed by a stage of model fitting. Systems such as DensePose^33^ and DenseBody^34^ provide segmentations that indicate correspondence between pixels and body parts of, or points on the surface of, the SMPL body model^35^. This is potentially very powerful data for fitting the model back to and recovering pose. A lack of cross camera consistency of the segmentations can make things difficult, and biomechanists may prefer a body model which has compatibility with the likes of OpenSim.

Directly inferring the parameters of a poseable body model from image data such as^36^ could also be explored with this dataset. Such systems are typically monocular and have to depend on prior knowledge to resolve ambiguity caused by the 2D to 3D solve. As well as evaluating how well the solution fits individual views, this dataset would allow testing consistency of the pose predictions between different camera views.

Most of the above paradigms involve a sequence of single-camera processing with later multi-camera fusion stages. That leaves the algorithms open to propagation of early errors throughout the workflow, which could have been avoided when using all viewpoints from the start. Volumetric and “early fusion” approaches^37–39^ to human pose estimation could also be evaluated with the BioCV dataset.

It is often necessary to fit a body model to the data extracted from the images, with biomechanics measurements then made from that model. To get the best possible fit of the model to the observations, it can help to know in advance the size and shape of the person being modelled. To this end, the BioCV dataset provides photogrammetry data with a view to this being used to tailor a body model to an individual prior to solving pose and motion.

Finally, the BioCV dataset provides a set of markerless trials for each individual. Researchers may wish to annotate this data and use it as training data to specialise their detectors to the dataset. The available data is only limited, but the availability of multiple viewpoints could help provide training data that have better viewpoint invariance than existing datasets.

### A Sparse Keypoints Markerless human pose pipeline

One possible approach^14^ to markerless human pose estimation is as follows.

Acquire and install the mc_reconstruction and mc_opensim repositories and their dependencies, as per their instructions. These tools are part of the code made available alongside the BioCV dataset (see the Code section). Furthermore, acquire and install OpenPose^28^.

The full process is then:

1. Use OpenPose to detect keypoints in each video of a motion trial
2. Use the trackSparsePoses tool of mc_reconstruction to perform cross-camera matching and person tracking using the keypoint detections and camera calibrations.
3. Use the fuseSparsePoses tool of mc_reconstruction to robustly estimate 3D coordinates for the keypoints using the cross-camera tracking information.
4. Use the tools of mc_opensim to fit an OpenSim model to the 3D keypoints.

Fuller documentation of this pipeline is available with the tools.

### Reference models for validation

To validate the performance of a markerless system, a suitable reference is first needed. No technology can give a true ground truth measurement of the positions of a person’s bones and muscles, but marker-based motion capture, as an accepted industry tool, makes an obvious reference.

However, even with marker trajectories, a full estimate of a person’s pose requires solving the parameters of a model that best suits the marker observations.

The mc_opensim repository provides the model files and fitting procedures for generating a reference OpenSim model used in publications using pre-release versions of the BioCV dataset.

## Data Availability

All of the custom software tools used for the creation of the BioCV dataset, as well as for many of the processing stages performed as part of related publications mentioned in the Summary Section, are available via Github at https://github.com/camera-mc-dev/.Of particular note will be the repositories mc_core, mc_reconstruction, mc_opensim, mc_annotator and mc_grabber which provide: mc_core: - camera calibration tools - core library functions like maths, renderers etc. mc_reconstruction: - cross-camera object association via occupancy maps - occupancy based object tracking - 3D reconstruction of sparse pose detections - motion capture visualisations * reprojection of markers onto images * reprojection of sparse pose reconstructions onto images * reprojection of OpenSim model fits onto images mc_opensim: - fit OpenSim model to motion capture marker trajectories - fit OpenSim model to markerless sparse pose reconstructions. mc_annotator - Tool for annotation of points in multi-camera image/video data mc_grabber: - data capture tool for SISO framegrabbers. All repositories contain full documentation for typical usage, as well as descriptions of algorithms where appropriate.

## References

[1] Colyer, S., Evans, M., Cosker, D. & Salo, A. A review of the evolution of vision-based motion analysis and the integration of advanced computer vision methods towards developing a markerless system. *Sports Medicine - Open* **4**, 1–15, 10.1186/s40798-018-0139-y (2018). 29869300 10.1186/s40798-018-0139-y PMC5986692

[2] Wade, L., Needham, L., McGuigan, P. & Bilzon, J. Applications and limitations of current markerless motion capture methods for clinical gait biomechanics. *Peer J* **10**, 10.7717/peerj.12995 (2022). 10.7717/peerj.12995 PMC8884063 35237469

[3] Leardini, A., Chiari, L., Croce, U. D. & Cappozzo, A. Human movement analysis using stereophotogrammetry: Part 3. soft tissue artifact assessment and compensation. *Gait & Posture* **21**, 212–225, 10.1016/j.gaitpost.2004.05.002 (2005). 15639400 10.1016/j.gaitpost.2004.05.002

[4] Khan, N. U. & Wan, W. A review of human pose estimation from single image. In *2018 International Conference on Audio, Language and Image Processing (ICALIP)*, 230–236, 10.1109/ICALIP.2018.8455796 (2018).

[5] Wang, J. *et al*. Deep 3d human pose estimation: A review. *Computer Vision and Image Understanding* **210**, 103225, 10.1016/j.cviu.2021.103225 (2021).

[6] Kumar, P., Chauhan, S. & Awasthi, L. Human pose estimation using deep learning: review, methodologies, progress and future research directions. *International Journal of Multimedia Information Retrieval* **11**, 1–33, 10.1007/s13735-022-00261-6 (2022).35096506

[7] Sigal, L., Balan, M. & Black, M. J. Humaneva: Synchronized video and motion capture dataset and baseline algorithm for evaluation of articulated human motion. *International Journal of Computer Vision* **4**, 10.1007/s11263-009-0273-6 (2010).

[8] Ionescu, C., Papava, D., Olaru, V. & Sminchisescu, C. Human3.6m: Large scale datasets and predictive methods for 3d human sensing in natural environments. *IEEE Transactions on Pattern Analysis and Machine Intelligence* **36**, 1325–1339, 10.1109/TPAMI.2013.248 (2014). 26353306 10.1109/TPAMI.2013.248

[9] Mundt, M., Born, Z., Goldacre, M. & Alderson, J. Estimating ground reaction forces from two-dimensional pose data: A biomechanics-based comparison of alphapose, blazepose, and openpose. *Sensors* **23**, 10.3390/s23010078 (2023). 10.3390/s23010078 PMC9823796 36616676

[10] Johnson, W. R., Mian, A., Lloyd, D. G. & Alderson, J. A. On-field player workload exposure and knee injury risk monitoring via deep learning. *Journal of Biomechanics* **93**, 185–193, 10.1016/j.jbiomech.2019.07.002 (2019). 31307769 10.1016/j.jbiomech.2019.07.002

[11] Pogson, M., Verheul, J., Robinson, M. A., Vanrenterghem, J. & Lisboa, P. A neural network method to predict task- and step-specific ground reaction force magnitudes from trunk accelerations during running activities. *Medical Engineering & Physics* **78**, 82–89, 10.1016/j.medengphy.2020.02.002 (2020). 32115354 10.1016/j.medengphy.2020.02.002

[12] Wade, L. *et al*. Examination of 2d frontal and sagittal markerless motion capture: Implications for markerless applications. *PLOS ONE* **18**, 1–16, 10.1371/journal.pone.0293917 (2023). 10.1371/journal.pone.0293917 PMC10635560 37943887

[13] Wade, L., Needham, L., McGuigan, P. & Bilzon, J. Backward double integration is a valid method to calculate maximal and sub-maximal jump height. *Journal of Sports Sciences* **40**, 1191–1197, 10.1080/02640414.2022.2059319 (2022). 35356858 10.1080/02640414.2022.2059319

[14] Needham, L. *et al*. The development and evaluation of a fully automated markerless motion capture workflow. *Journal of Biomechanics* **144**, 10.1016/j.jbiomech.2022.111338 (2022). 10.1016/j.jbiomech.2022.111338 36252308

[15] Needham, L. *et al*. The accuracy of several pose estimation methods for 3d joint centre localisation. *Scientific Reports* **11**, 10.1038/s41598-021-00212-x (2021). 10.1038/s41598-021-00212-x PMC8526586 34667207

[16] Needham, L. *et al*. Development and evaluation of a deep learning based markerless motion capture system. In *International Conference on Biomechanics in Sports, ISBS 2021*, http://www.isbs2021.org/ (2021).

[17] Needham, L. *et al*. The performance of open-source pose estimation algorithms during walking, running and jumping. In *Congress of the International Society of Biomechanics* (2021).

[18] Evans, M. & Needham, L. mc-dev: C++ experiments in multi-camera and markerless motion capture. https://github.com/camera-mc-dev.

[19] Bradski, G. The OpenCV Library. *Dr. Dobb’s Journal of Software Tools* (2000).

[20] Zhang, Z. A flexible new technique for camera calibration. *IEEE Transactions on Pattern Analysis and Machine Intelligence* **22**, 1330–1334, 10.1109/34.888718 (2000).

[21] Agarwal, S., Mierle, K. & The Ceres Solver Team. Ceres Solver https://github.com/ceres-solver/ceres-solver (2022).

[22] Triggs, B., McLauchlan, P. F., Hartley, R. I. & Fitzgibbon, A. W. Bundle adjustment — a modern synthesis. In *Vision Algorithms: Theory and Practice: International Workshop on Vision Algorithms Corfu, Greece, September 21–22, 1999 Proceedings*, 298–372 (Springer Berlin Heidelberg, Berlin, Heidelberg, 10.1007/3-540-44480-7_21 2000).

[23] Bell, A. L., Brand, R. A. & Pedersen, D. R. Prediction of hip-joint center location from external landmarks. *Human Movement Science* **8**, 3–16, 10.1016/0167-9457(89)90020-1 (1989).

[24] Stanhope, S. *et al*. Kinematic-based technique for event time determination during gait. *Medical and Biological Engineering and Computing* **28**, 355–360, 10.1007/BF02446154 (1990). 2246935 10.1007/BF02446154

[25] Evans, M. *et al*. biocv motion capture dataset. *University of Bath Research Data Archive*10.15125/BATH-01258 (in press)

[26] Michaud, B. & Begon, M. ezc3d: An easy c3d file i/o cross-platform solution for C++, Python and MATLAB. *Journal of Open Source Software* **6**, 2911, 10.21105/joss.02911 (2021).

[27] Bouguet, J.-Y. *Camera Calibration Toolbox for Matlab* (2022).

[28] Cao, Z., Hidalgo, G., Simon, T., Wei, S. & Sheikh, Y. Openpose: Realtime multi-person 2d pose estimation using part affinity fields. *IEEE Transactions on Pattern Analysis &; Machine Intelligence* **43**, 172–186, 10.1109/TPAMI.2019.2929257 (2021). 31331883 10.1109/TPAMI.2019.2929257

[29] Fang, H.-S., Xie, S., Tai, Y.-W. & Lu, C. Rmpe: Regional multi-person pose estimation. In *2017 IEEE International Conference on Computer Vision (ICCV)*, 2353–2362, 10.1109/ICCV.2017.256 (2017).

[30] Contributors, M. Openmmlab pose estimation toolbox and benchmark. https://github.com/open-mmlab/mmpose (2020).

[31] Uhlrich, S. D. *et al*. Opencap: Human movement dynamics from smartphone videos. *PLOS Computational Biology* **19**, 1–26, 10.1371/journal.pcbi.1011462 (2023). 10.1371/journal.pcbi.1011462 PMC10586693 37856442

[32] Pagnon, D., Domalain, M. & Reveret, L. Pose2sim: An open-source python package for multiview markerless kinematics. *Journal of Open Source Software*10.21105/joss.04362 (2022).

[33] Güler, R. A., Neverova, N. & Kokkinos, I. Densepose: Dense human pose estimation in the wild. In *Proceedings of the IEEE Conference on Computer Vision and Pattern Recognition*, 7297–7306, 10.1109/CVPR.2018.00762 (2018).

[34] Yao, P., Fang, Z., Wu, F., Feng, Y. & Li, J. Densebody: Directly regressing dense 3d human pose and shape from a single color image 1903.10153, 10.48550/arXiv.1903.10153 (2019).

[35] Loper, M., Mahmood, N., Romero, J., Pons-Moll, G. & Black, M. J. SMPL: A skinned multi-person linear model. *ACM Trans. Graphics (Proc. SIGGRAPH Asia)* **34**, 248:1–248:16, 10.1145/2816795.2818013 (2015).

[36] Kanazawa, A., Black, M., Jacobs, D. & Malik, J. End-to-end recovery of human shape and pose. In *Computer Vision and Pattern Regognition (CVPR)*, 7122–7131, 10.1109/CVPR.2018.00744 (2018).

[37] Varol, G. *et al*. Bodynet: Volumetric inference of 3d human body shapes. In Ferrari, V., Hebert, M., Sminchisescu, C. & Weiss, Y. (eds.) *Computer Vision – ECCV 2018*, 20–38, 10.1007/978-3-030-01234-22 (Springer International Publishing, Cham, 2018).

[38] Tu, H., Wang, C. & Zeng, W. Voxelpose: Towards multi-camera 3d human pose estimation in wild environment. In Vedaldi, A., Bischof, H., Brox, T. & Frahm, J.-M. (eds.) *Computer Vision – ECCV 2020*, 197–212, 10.1007/978-3-030-58452-812 (Springer International Publishing, Cham, 2020)

[39] Lin, J. & Lee, G. H. Multi-view multi-person 3d pose estimation with plane sweep stereo. In *2021 IEEE/CVF Conference on Computer Vision and Pattern Recognition (CVPR)*, 11881–11890, 10.1109/CVPR46437.2021.01171 (2021).

